# Salivary Fingerprint in the Metabolomics Era: Potential and Challenges

**DOI:** 10.3390/metabo15080545

**Published:** 2025-08-12

**Authors:** Tatiana Kelly da Silva Fidalgo, Ana Paula Valente

**Affiliations:** 1 Department of Preventive and Community Dentistry, Dental School, Rio de Janeiro State University, Boulevard 28 de Setembro 157, Vila Isabel, Rio de Janeiro 20551-030, Rio de Janeiro, Brazil; 2 National Centre of Nuclear Magnetic Resonance-CENABIO, Institut of Medical Biochemistry, Federal University of Rio de Janeiro, Rio de Janeiro 21941-590, Rio de Janeiro, Brazil

## 1. Introduction

In recent years, the field of metabolomics has significantly advanced our understanding of human health and disease by providing a comprehensive snapshot of metabolic alterations. Among the various biofluids used in metabolomic investigations, saliva has emerged as a non-invasive, accessible, and information-rich source of biochemical data. Its ease of collection and the growing number of salivary biomarkers linked to systemic and local diseases make it an attractive candidate for precision medicine [1].

The concept of a “salivary fingerprint” refers to a unique, dynamic metabolic profile reflective of individual physiological states, and presents great promise in enabling personalized diagnostics and health monitoring. However, several technical, biological, and translational challenges continue to hinder its widespread implementation in clinical practice.

Why saliva? Saliva secretion is a complex and dynamic biofluid primarily composed of water, but it also contains a wide array of proteins, electrolytes, nucleic acids, hormones, and microorganisms, as well as a diverse range of metabolites. Secreted by major and minor salivary glands, its composition reflects both local oral processes and systemic physiological conditions. Saliva is secreted into the oral cavity, where it mixes with gingival crevicular fluid, microorganisms and their metabolic products, and exogenous substances such as food, medications, and oral care products, forming what is known as whole saliva. The salivary glands are richly vascularized, and the crevicular fluid often contains inflammatory infiltrates that reflect systemic immune activity. These characteristics allow saliva to capture both local and systemic physiological changes, making it a valuable diagnostic fluid, especially in contexts where non-invasive, painless, and repeatable sampling is essential [2,3,4,5].

From a practical standpoint, saliva collection offers several advantages over traditional matrices like blood or urine. It does not require trained personnel, sterile environments, or invasive procedures. This simplicity enables repeated sampling in a variety of settings, including in pediatric, geriatric, or critically ill populations. Moreover, saliva can be collected with minimal discomfort and low biohazard risk, features that are especially relevant for population-based studies [2].

Over the past decade, research on the salivary metabolome has grown substantially, revealing an increasing number of metabolites linked to both local and systemic health conditions. Saliva contains hundreds of small molecules—such as amino acids, organic acids, lipids, and carbohydrates—many of which are also present in blood, albeit at significantly lower concentrations.

NMR is highly reproducible and non-destructive. In addition, saliva samples require minimal preparation, and a single analytical run can detect over 200 distinct molecules; however, they suffer from limited sensitivity, which can be problematic given the relatively low metabolite concentrations in saliva. In contrast, LC-MS and GC-MS offer higher sensitivity, but they require more critical sample preparation.

## 2. Challenges

Despite these benefits, saliva presents significant analytical and biological challenges that complicate its use in metabolomics. Its composition is highly susceptible to the consumption of food or drink, the presence of microorganisms, and environmental factors. Flow rate and pH can vary significantly depending on the method of collection, stimulated vs. unstimulated, diurnal cycle, and hydration status. These variables can affect both the concentration and detectability of salivary metabolites, contributing to inter- and intra-individual variability [6,7].

Furthermore, salivary metabolite concentrations are lower than those found in blood or urine, requiring highly sensitive analytical techniques and robust sample preparation protocols. The absence of standardized protocols for saliva collection, storage, and processing across studies hampers the reproducibility and comparability of results, thereby limiting the reliability of findings. Therefore, pre-analytical variables, such as collection method, time of day, and storage conditions, must be rigorously controlled to reduce noise and improve data quality. Moreover, standardized workflows tailored to saliva-specific metabolomics require further refinement to enable robust cross-study comparisons and support biomarker validation efforts [8]. These inconsistencies impact metabolite stability and data comparability across studies.

Another challenge is the acquisition of robust longitudinal data. Most current studies are cross-sectional, making it difficult to define stable individual fingerprints or understand intra-individual variability over time. Well-established longitudinal baselines are necessary to determine whether a given metabolic shift is pathological or simply a normal fluctuation. Therefore, several challenges must be addressed for salivary metabolomics to transition from research to real-world application.

As in any other analytical technique, salivary metabolomics is susceptible to the risk of false negatives or data loss. Advanced instrumentation and rigorous sample processing are required to mitigate these effects. A significant portion of salivary metabolites originates not from the host, but from the oral microbiota. While this may provide additional diagnostic value, it also complicates efforts to interpret host-specific metabolic changes.

## 3. Opportunities

Despite limitations, salivary metabolomics offers promising opportunities for non-invasive diagnostics, disease monitoring, and personalized health strategies. Although still in development, advances in analytical technologies and portable health devices are bringing saliva-based applications nearer to clinical implementation.

The notion of a “salivary fingerprint” refers to the idea that an individual’s salivary metabolome reflects a unique and dynamic profile shaped by their physiological, pathological, lifestyle, and environmental factors. This personalized metabolic signature is potentially valuable for longitudinal health monitoring, early disease detection, and guiding individualized interventions, all key elements of precision medicine.

In oral health, saliva demonstrated significants modifications with local diseases, such as dental caries and periodontal disease. Conditions such as periodontitis, dental caries, and oral squamous cell carcinoma exhibit clearer metabolic signatures, partly due to the direct contact of saliva with the affected tissues [7,9]. Given the impact of oral alterations on salivary metabolomics, it is essential to identify the metabolites associated with these diseases. Moreover, for multifactorial oral diseases such as dental caries and periodontal disease, the primary objective is not merely biodiagnosis, but rather a comprehensive understanding of the physiological changes occurring within the salivary biofluid [10,11].

Numerous studies have demonstrated that saliva can reflect systemic metabolic alterations. For example, altered salivary metabolite profiles have been reported in patients with type 2 diabetes, renal failure, cardiovascular disease, and various cancers, including oral, breast, and pancreatic cancer. In neurodegenerative diseases like Alzheimer’s and Parkinson’s, preliminary findings suggest that salivary metabolites may provide non-invasive diagnostic insights, although validation in larger cohorts remains lacking [1,2,3,12,13]. In chronic and degenerative disease management, saliva enables frequent, comfortable sampling, making it suitable for monitoring treatment response, metabolic fluctuations, or stress levels.

In theory, such a fingerprint could enable non-invasive tracking of subtle metabolic shifts before clinical symptoms emerge. For example, an individual’s baseline salivary profile could serve as a reference, with deviations indicating inflammatory processes, metabolic dysregulation, or psychological stress. In chronic disease management, salivary fingerprints could help assess treatment response or disease progression in real time.

The current Special Issue, “Salivary Fingerprint in the Metabolomics Era: Potential and Challenges”, includes a diverse range of studies exploring physiological conditions, such as those occurring during early developmental stages and aging, as well as acute conditions like SARS-CoV-2 infection and inflammation, and chronic diseases including cancer, obesity, and periodontal disease [14]. Together, these contributions underscore the growing relevance of salivary metabolomics as a non-invasive and promising tool in both research and, potentially, clinical practice. Looking forward, the concept of a personalized “salivary fingerprint” could be integrated into precision medicine frameworks. However, significant steps are required to reach clinical implementation, including standardized protocols, large-scale validation, and integration with other omics and clinical data.

While saliva is unlikely to replace traditional matrices such as blood, its unique advantages position it as a valuable complementary tool in future healthcare. With strategic investment, interdisciplinary collaboration, and a strong emphasis on reproducibility and usability, salivary metabolomics has the potential to evolve from an experimental approach into a practical asset for both clinical practice and public health applications.

## 4. Conclusions

Salivary metabolomics holds significant promise for non-invasive diagnostics, disease monitoring, and personalized medicine. While challenges such as biological variability, methodological inconsistencies, and limited clinical validation remain, advances in technology and standardization offer a path forward. With continued research, interdisciplinary collaboration, and robust clinical trials, saliva could complement traditional biofluids in healthcare, offering a cost-effective, accessible tool for early disease detection and ongoing health monitoring. The journey from concept to clinical application is complex, but the potential rewards make it a worthwhile pursuit for the future of precision medicine.
